# Antioxidant Efficacy of Olive By-Product Extracts in Human Colon HCT8 Cells

**DOI:** 10.3390/foods10010011

**Published:** 2020-12-23

**Authors:** Mariangela Centrone, Mariagrazia D’Agostino, Graziana Difonzo, Alessandra De Bruno, Annarita Di Mise, Marianna Ranieri, Cinzia Montemurro, Giovanna Valenti, Marco Poiana, Francesco Caponio, Grazia Tamma

**Affiliations:** 1 Department of Biosciences, Biotechnologies and Biopharmaceutics, University of Bari Aldo Moro, 70125 Bari, Italy; mariangela.centrone@uniba.it (M.C.); mariagrazia.dagostino@uniba.it (M.D.); annarita.dimise@uniba.it (A.D.M.); marianna.ranieri@uniba.it (M.R.); giovanna.valenti@uniba.it (G.V.); 2 Department of Soil, Plant and Food Sciences, University of Bari Aldo Moro, 70125 Bari, Italy; graziana.difonzo@uniba.it (G.D.); cinzia.montemurro@uniba.it (C.M.); francesco.caponio@uniba.it (F.C.); 3 Department of AGRARIA, University Mediterranea of Reggio Calabria, Vito, 89124 Reggio Calabria, Italy; alessandra.debruno@unirc.it (A.D.B.); mpoiana@unirc.it (M.P.)

**Keywords:** olive by-product, reactive oxygen species (ROS), olive leaf, pomace, olive wastewater

## Abstract

The production of olive oil is accompanied by the generation of a huge amount of waste and by-products including olive leaves, pomace, and wastewater. The latter represents a relevant environmental issue because they contain certain phytotoxic compounds that may need specific treatments before the expensive disposal. Therefore, reducing waste biomass and valorizing by-products would make olive oil production more sustainable. Here, we explore the biological actions of extracts deriving from olive by-products including olive pomace (OP), olive wastewater (OWW), and olive leaf (OLs) in human colorectal carcinoma HCT8 cells. Interestingly, with the same phenolic concentration, the extract obtained from the OWW showed higher antioxidant ability compared with the extracts derived from OP and OLs. These biological effects may be related to the differential phenolic composition of the extracts, as OWW extract contains the highest amount of hydroxytyrosol and tyrosol that are potent antioxidant compounds. Furthermore, OP extract that contains a higher level of vanillic acid than the other extracts displayed a cytotoxic action at the highest concentration. Together these findings revealed that phenols in the by-product extracts may interfere with signaling molecules that cross-link several intracellular pathways, raising the possibility to use them for beneficial health effects.

## 1. Introduction

Olive cultivation is typical of Mediterranean countries and it is developed in several regions of the world having similar climate features [1]. By-products and waste from olive production and the olive oil industry represent a relevant environmental issue because they contain several compounds that may also be phytotoxic [2]. Olive wastewater (OWW) contains a huge bulk of organic compounds, metals, minerals including potassium, calcium, and phosphorous. Furthermore, OWW has high biological and chemical oxygen demand (BOD and COD) and very low pH that make this olive waste highly pollutant [3]. In developed countries, the growing demand for food increases the mass of waste and by-products in an unsustainable manner. In the last few years, novel approaches, based on the new concept of the circular economy, lead to reduce waste biomass and valorize by-products. The improvement of extraction strategies provides a better exploit of olive by-products for functional food production, the development of pharmaceutical supplements and, cosmeceuticals [4]. Along the olive oil chain, olive mills produce tons of by-products including olive pomace (OP), olive mill wastewater (OWW), and olive leaves (OLs). These latter are commonly used for animal feeding or direct combustion. Recent studies report that OLs contain certain bioactive compounds that may be useful for the preparation of nutraceuticals and supplements.

OP contains pulp, skin, stone and water. However, the chemical composition and the biological activity of OP may differ according to the oil extraction method. Pomace is mainly used to produce pomace olive oil and a more recent application includes the generation of fuel after microbiological fermentation processes [5]. In contrast, the disposal of the olive mill wastewater is still an expensive cost for the oil olive industry. Notably, recent reports propose new methods based on nanocentrifugation and ultrafiltration to revalorize the olive wastewater [6,7]. Chemical characterization studies revealed that some bioactive compounds are transferred from olives to oil and their by-products and waste during the oil production process [8]. Specifically, OP, OWW, and OLs contain bioactive compounds including secoiridoids, squalene, flavonoids, lignans, phytosterols, tocopherols, and phenols. Among them, phenols, which displayed a significant antioxidant capability, are extensively investigated in different fields of research. The highest bulk of phenols have been isolated in olive wastewater. By contrast, a low amount of phenols was detected in the oil [9]. Numerous studies investigated the functional involvement of phenols in human health and healthy aging. The biological actions of phenols may be due to their antioxidant and anti-inflammatory properties. Nevertheless, the molecular mechanism of action of these compounds, on different diseases, is still unclear because they may have multiple intracellular targets. In bronchial epithelial NCI-H292 cells, a green olive leaf extract (OLE), obtained using water as an extraction solvent, reduced the tBHP-induced reactive oxygen species (ROS) production. This extract indeed displayed a significant antioxidant capability in vegetable oil [10]. Furthermore, in renal collecting duct MCD4 cells, the green OLE counteracted the cytotoxic effects due to long exposure to low doses of cadmium [11]. Specifically, in cells exposed to cadmium, OLE reduced the frequency of micronuclei, DNA double-strand breaks, and anomalous alterations of the cytoskeleton by modulating the S-glutathionylation of actin [11]. Therefore, OLE may exert different cellular responses that may be only partially due to antioxidant features. The olive derived extracts and by-products contain numerous compounds such as hydroxytyrosol and oleuropein that can modulate different intracellular signal transduction pathways including the ones regulating autophagy and apoptosis [12,13]. Chronic and acute disturbances affecting the gastrointestinal tract such as inflammatory bowel diseases are associated with an abnormal intracellular level of reactive species due to a relevant unbalance between the ROS production systems and the antioxidant intestinal defenses [14]. Moreover, colonic mucosa of patients with chronic inflammatory diseases produced a high amount of ROS compared with normal mucosa [15]. Therefore, the administration of selective antioxidant phytocompounds may provide beneficial effects to counteract intracellular processes leading to abnormal production of ROS in the intestine. In the present study, the human colon HCT8 cells were used as an experimental model to assay the cell viability and the antioxidant activity of extracts deriving from olive by-products including OP, OWW, and OLs. HCT-8 cell line derived from the ileocecal colon displays a high proliferation and migration rate associated with high ROS production [16]. Moreover, compared with the fully differentiated HT-29 and Caco-2 colon cell lines, HCT-8 cells have poorly organized junctional complexes [16,17]. Interestingly, here, with the same phenolic concentration, the extract obtained from the olive wastewater displayed a higher antioxidant activity compared with the extracts derived from olive pomace and leaves.

## 2. Materials and Methods

### 2.1. Chemicals and Reagents

Cell culture media and FBS (fetal bovine serum) were from GIBCO (Thermo Fisher Scientific, Waltham, MA, USA). Dihydrorhodamine-123 was obtained from Invitrogen™ (Thermo Fisher Scientific, Waltham, MA, USA). The tert-Butyl hydroperoxide (tBHP) was a kind gift from A. Signorile (University of Bari Aldo Moro, Bari, Italy).

### 2.2. Extraction of Phenolic Compounds from By-Products

#### 2.2.1. Olive Wastewater (OWW)

OWW was collected during the crop seasons 2019/2020 from Ottobratica olive cultivar and produced according to a three-phase centrifugation process. The phenolic extract was obtained following the method reported by Romeo et al. [18] with some modifications. Two liters of OWW was acidified to pH 2 with HCl and washed three times with hexane (1:1, *v*/*v*) to remove the lipid fraction. The mixture was vigorously shaken and centrifuged under 3000 rpm for 3 min at 10 °C. The extraction procedure was carried out using ethyl acetate three times and the solvent was recovered in a separating funnel (1:4, *v*/*v*). The two-phases (water and ethyl acetate) were separated and both were evaporated using a rotary vacuum evaporator at 25 °C. Finally, the dry residues were again dissolved in 100 mL of water, filtered using PTFE 0.45 μm (diameter 15 mm) syringe filter, and stored at 4 °C until subsequent analyses.

#### 2.2.2. Olive Pomace (OP)

OP was collected during the crop seasons 2019/2020 from Ottobratica olive cultivar and produced according to a three-phase centrifugation process. The phenolic extract was obtained following the method reported by De Bruno et al. [19] with some modifications. 200 g of defatted olive pomace was extracted by way ultrasound system (W%, 15 OFF 5 s) with ethanol/water 80/20 for 1 h. The extract was filtered using Buchner funnel and evaporated to 80% in a rotary evaporator at 25 °C.

#### 2.2.3. Olive Leaves (OLs)

The olive leaves were collected in the crop seasons 2019/2020 from Coratina olive cultivar, stored at 4 °C, and processed in less than 24 h. The extraction was performed according to Difonzo et al. [10]. After washing with tap water at room temperature, the olive leaves were dried at 120 °C for 8 min in a ventilated oven (Argolab, Carpi, Italy) to reach a moisture content <1%, milled with a blender (Waring-Commercial, Torrington, CT, USA). The extraction from leaves was ultrasound-assisted (CEIA, Viciomaggio, Italy) and water was added in a ratio 1/20 (*w*/*v*). Three washes were done, each one for 30 min at 35 ± 5 °C. Finally, the extracts were filtered through Whatman (GE Healthcare, Milan, Italy) filter paper, freeze-dried, and stored at −20 °C. Before the analysis, the extract was filtered by nylon filters of 0.45 µm.

### 2.3. Chemical Characterization of Extracts

#### 2.3.1. Olive Wastewater (OWW) and Olive Pomace (OP)

The total phenol content (TPC) and antioxidant activity (ABTS, 2,2′-azino bis(3 ethylbenzothiazoline-6-sulfonic acid)) were determined spectrophotometrically following the method described by De Bruno [19] with some modifications. TPC, was quantified on the obtained extracts by FolinCiocalteau method, 0.1 mL of the phenolic extract (OWW and OP), were placed in a 25 mL volumetric flask and mixed with 20 mL of deionized water and 0.625 mL of the FolinCiocalteau reagent. After 3 min, 2.5 mL of a saturated solution of Na_2_CO_3_ (20%) were added. The content was mixed and diluted to volume with deionized water. Thereafter the mixture was incubated for 12 h at room temperature and in the dark. The absorbance of the samples was measured at 725 nm against a blank using a double-beam ultraviolet-visible spectrophotometer (Perkin-Elmer UV-Vis λ2, Waltham, MA, USA) and comparing with a gallic acid calibration curve (concentration between 1 and 10 mg·L^−1^). The results were expressed as mg of GAE mL^−1^. For total antioxidant activity determination, ABTS assay was used. The reaction mixture was prepared by mixing 2990 µL of ABTS and 10 µL of extracts (OWW and OP), and the absorbance was measured after 6 min at 734 nm. The quenching of initial absorbance was plotted against the Trolox concentration (from 1.5 to 24 mmol L^−1^) and the TEAC value was expressed as µmol TE mL^−1^ of PE. Identification and determination of the main bioactive phenolic compounds were performed by UHPLC-DAD of the phenolic extract, following the method described by Romeo et al. [18] with some modifications. The UHPLC system consisted of an UHPLC PLATINblue (Knauer, Berlin, Germany) equipped with a binary pump system using a Knauer blue orchid column C18 (1.8 μm, 100 × 2 mm) coupled with a PDA-1 (Photo Diode Array Detector) PLATINblue (Knauer, Berlin, Germany). The used software was Clarity 6.2 (Clarity-DataApex, Prague, The Czech Republic). The samples were filtered with a 0.22 μm nylon syringe filters (diameter 13 mm) and then injected in the system with a volume of 5 μL. The mobile phases were (A) water acidified with acetic acid (pH 3.10) and (B) acetonitrile; the gradient elution program consisted of 0–3 min, 95% A; 3–15 min, 95%–60% A; 15–15.5 min, 60%–0% A; finally, returning to the initial conditions was achieved during analysis keeping the column at 30 °C and the injection volume 5 μL. External standards (concentration between 1 and 100 mg·L^−1^) were used for the quantification and the results were expressed as mg·mL^−1^.

#### 2.3.2. Olive Leaves (OLs)

Total phenol content, antioxidant activity, and single phenolic compounds identification were performed according to Difonzo et al. [10] with some modifications. For the total phenol content determination, 20 µL of the extract was added to 980 µL of ddH_2_O and 100 µL of FolinCiocalteu reagent. After 3 min, 5% Na_2_CO_3_ solution was added, following incubation at room temperature for 60 min. The absorbance was read at 750 nm using a Cary 60 spectrophotometer (Agilent, Milan, Italy). The TPC was expressed as gallic acid equivalents (GAE) in mg·L^−1^ juice.

For the antioxidant activity assay ABTS, the radical was generated by a chemical reaction with potassium persulfate (K_2_S_2_O_8_). For this purpose, 25 mL of ABTS (7 mM in H_2_O) was spiked with 440 μL of K_2_S_2_O_8_ (140 mM) and allowed to stand in darkness at room temperature for 12–16 h (the time required for the formation of the radical). The working solution was prepared by taking a volume of the previous solution and diluting it in ethanol until its absorbance at *λ* = 734 nm was 0.70 ± 0.02. A Cary 60 spectrophotometer Agilent (Milan, Italy) was used. The reaction took place directly in the measuring cuvette: 50 µL of each sample was added to 950 µL of the final ABTS**^·^**^+^ solution. After 8 min the decrease of absorbance was measured at 734 nm. The results are expressed in μmol Troloxequivalents (TE) g^−1^ dry weight. Each sample was analyzed in triplicate.

The main identified phenolic compounds were quantified by means of HPLC-DAD (Dionex Ultimate 3000 RSLC, Waltham, MA, USA). Dionex Acclaim 120 C18 analytical column (150 × 3 mm i.d.) with a particle size of 3 μm (Thermo Scientific, Waltham, MA, USA) was used. The mobile phases were (A) water/acetic acid (98:2, *v*/*v*) and (B) acetonitrile at a constant flow rate of 1 mL·min^−1^. The column temperature was set at 35 °C. The gradient program was as follows: 5 min, 95% A; 10 min, 80% A; 15 min, 75% A; 20 min, 65% A; 25 min, 0% A; 35 min, 95% A. The identification of phenolic compounds was based on a comparison of retention times obtained by pure standard (Sigma-Aldrich Co. LLC, St. Louis, MO, USA) and literature data [10]. The quantification was carried out by means of external calibration curves with the relative standard for the selected compounds.

### 2.4. Cell Culture and Treatment

The human colon cancer cells HCT-8 were cultured as previously described [20]. Briefly, cells were grown in Advanced RPMI-1640 supplemented with 10% fetal bovine serum (FBS), 100 i.u. mL^−1^ penicillin, 100 µg·mL^−1^ streptomycin at 37 °C in 5% CO_2_. Cells were left under basal condition or treated overnight with the same phenolic concentration (0.03 mg·L^−1^, 0.06 mg·L^−1^, and 0.12 mg·L^−1^) obtained from extracts of olive leaves or olive pomace or olive mill wastewater.

### 2.5. Crystal Violet Assay

Crystal violet assay was performed as previously described [11]. Briefly, cells were grown in a 96-well plate and left under basal condition or stimulated as mentioned before. Cells were fixed with 4% paraformaldehyde for 20 min and then stained with a solution containing 0.1% crystal violet for 20 min. After washing, cells were lysed with 10% acetic acid. The optical density at 595 nm (DO595) of each well was measured with a Microplate Reader (Bio-Rad Laboratories, Inc., Hercules, CA, USA) and was used as a measurement of cell viability.

### 2.6. Reactive Oxygen Species (ROS) Detection

ROS were detected as already shown [11]. After treatments, cells were incubated with dihydrorhodamine-123 (10 μM) for 30 min at 37 °C with 5% CO_2_ and recovered in complete medium for 30 min. Cells were lysed in RIPA buffer containing 150 mM NaCl, 10 mM Tris-HCl pH 7.2, 0.1% SDS, 1.0% Triton X-100, 1% sodium deoxycholate and 5 mM EDTA. Samples were centrifuged at 12,000× *g* for 10 min at 4 °C and the supernatants were used for ROS detection. As a positive control, cells were treated with tert-Butyl hydroperoxide (tBHP, 2 mM for 30 min). The fluorescence emission signal was recorded using a fluorimeter (RF-5301PC, Shimadzu Corporation, Kyoto, Japan) at excitation and emission wavelengths of 508 and 529 nm, respectively.

### 2.7. Statistical Analysis

All values are reported as means ± S.E.M. Statistical analysis was performed by one-way ANOVA followed by Dunnett’s multiple comparisons test with * *p* < 0.05 considered statistically different.

## 3. Results

### 3.1. Characterization of Olive By-Products and Waste

The valorization of waste and by-products derived from the olive oil industry represents an attractive and sustainable challenge aiming to reduce the environmental impact and the disposal costs. Extracts derived from by-products can contain several compounds displaying bioactive activity. The phenolic compounds recovery from waste and by-products is usually carried out using organic solvent extraction. The choice of the solvent to use is made according to the purpose of extraction, the polarity of the interesting components, overall cost, safety, etc. For this reason, in this study, the recovery of phenolic compounds was realized with hydro-alcoholic solutions for OP and OLs and ethyl acetate for OWW. The latter solvent showing a high extraction efficiency for phenolic extraction from OWW, and at the end of the extraction procedure is eliminated (through concentration), and the phenolic compounds were recovered with water.

Table 1 reports the results of total phenol content, antioxidant activity, and the chemical characterization of the main phenolics in the considered olive waste and by-products. Extracts obtained from OWW showed higher total phenolic content (about 9 mg·mL^−1^) compared to the pomace extract (4.5 mg·mL^−1^).

Among these, the main ones were: hydroxytyrosol, tyrosol, and oleuropein in agreement with our previous work [21]. Particularly, the OWW was rich in hydroxytyrosol (1.4 mg·mL^−1^), also if this content may vary according to several factors as oil extraction technique, OWW characteristics, cultivar, extraction method of phenolic content. Furthermore, for OP extract, the main phenolic compound was hydroxytyrosol, but in a lower amount (0.2 mg·mL^−1^). OWW extract showed a notably higher value of antioxidant activity compared to OP extract (3579 and 451 µmol TE mL^−1^, respectively), denoting a strong antioxidant activity against ABTS radical.

The OLE showed high total phenol content and antioxidant activity according to Ghasemi et al. [22]. Among phenols, oleuropein, verbascoside, apigenin-7-O-glucoside, luteolin-7-O-glucoside, tyrosol, and rutin were identified.

The most represented compounds were oleuropein with a concentration of 137 mg g^−1^ in line with the results of other authors [22,23], in fact, generally, the amount of this secoiridoid is in the range of 25–30% of the total polyphenols in OLE [24].

### 3.2. Biological Characterization of Olive By-Products

**Figure 1:**
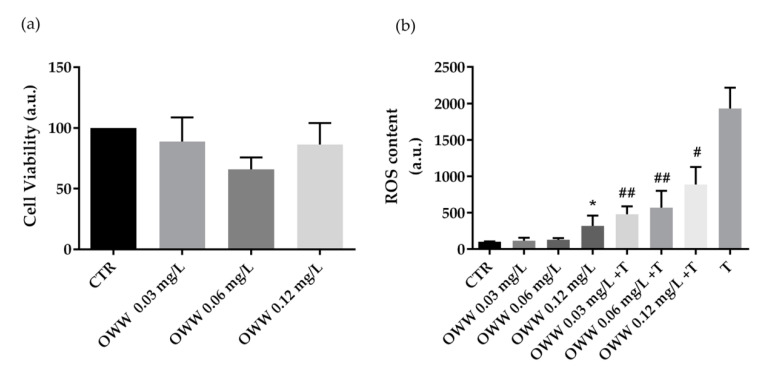
Cell viability (**a**) and ROS content (**b**) in HCT8 cells exposed to olive wastewater (OWW). (**a**) Cells were left under basal condition or exposed to increasing concentrations (0.03 mg·L^−1^, 0.06 mg·L^−1^, and 0.12 mg·L^−1^ of phenols) of OWW and were stained with crystal violet solution. Data are shown as means ± Standard Error Mean (S.E.M.) of 3 independent experiments and analyzed by one-way ANOVA followed by Dunnett’s multiple comparisons test. (**b**) ROS content was measured using dihydrorhodamine-123 fluorescence in cells treated as previously described. As a positive control, cells were treated with tert-butyl hydroperoxide (tBHP). Data are shown as means ± S.E.M. of 3 independent experiments and analyzed by one-way ANOVA followed by Dunnett’s multiple comparisons test. (* *p*< 0.05 vs. CTR; ## *p* < 0.01 and # *p* < 0.05 vs. tBHP).

An increase in systemic oxidative stress related to abnormal production of ROS is often associated with aging and several diseases including hypertension, obesity, and cancer. A growing number of bioactive phytocompounds, known for their beneficial and antioxidant actions on human health, are more frequently recommended as therapeutic adjuvants in several diseases [25]. Here, we evaluated the effects of extracts obtained from olive by-products and wastes on cell viability in human colorectal carcinoma HCT8 cells. Furthermore, the potential antioxidant efficacy of olive by-products and wastes was assayed as well. Cells were incubated overnight with extracts derived from OWW at increasing concentrations (0.03 mg·L^−1^, 0.06 mg·L^−1^, and 0.12 mg·L^−1^ of phenols). Compared to cells left under control conditions, treatment with OWW extracts does not alter cell viability at the concentration used (Figure 1a). Under similar conditions, ROS content was measured in HCT8 cells. Compared to untreated cells (CTR), ROS production increased in cells incubated with OWW extracts at 0.12 mg·L^−1^ (Figure 1b). Indeed, compared to the positive control (tBHP), cells co-incubated with the oxidant tBHP, and OWW extracts showed a significant decrease in ROS generation induced by tBHP (Figure 1b).

**Figure 2:**
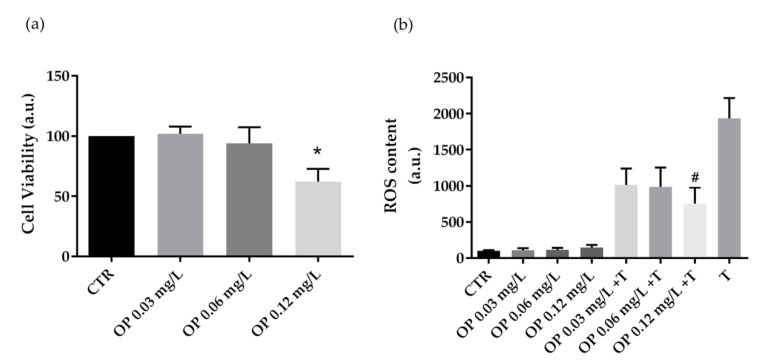
Cell viability (**a**) and ROS content (**b**) in HCT8 cells exposed to olive pomace (OP). (**a**) Cells were left under basal condition or exposed to increasing concentrations (0.03 mg·L^−1^, 0.06 mg·L^−1^, and 0.12 mg·L^−1^ of phenols) of OP and were stained with crystal violet solution. Data are shown as means ± S.E.M. of 3 independent experiments and analyzed by one-way ANOVA followed by Dunnett’s multiple comparisons test (* *p* < 0.05 vs. CTR). (**b**) ROS content was measured using dihydrorhodamine-123 fluorescence in cells treated as previously described. As a positive control, cells were treated with tert-butyl hydroperoxide (tBHP). Data are shown as means ± S.E.M. of 3 independent experiments and analyzed by one-way ANOVA followed by Dunnett’s multiple comparisons test. (# *p* < 0.05 vs. tBHP).

Next, HCT8 cells were treated with extracts obtained from OP at increasing concentrations (0.03 mg·L^−1^, 0.06 mg·L^−1^ and 0.12 mg·L^−1^ of phenols). Compared to CTR cells, treatment with OP extracts at 0.12 mg·L^−1^ of phenols displayed a cytotoxic effect (Figure 2a). Fluorimetric measurements revealed that cells co-incubated with the oxidant tBHP and OP extracts at 0.12 mg·L^−1^ of phenols showed a significant decrease in ROS generation induced by tBHP. However, a relevant but not significant reduction in ROS content was measured in cells co-treated with tBHP and OP extracts at 0.03 mg·L^−1^ and 0.06 mg·L^−1^ of phenols. Incubation with the OP extracts alone, at increasing concentrations (0.03 mg·L^−1^, 0.06 mg·L^−1^, and 0.12 mg·L^−1^ of phenols) does not alter ROS production.

**Figure 3:**
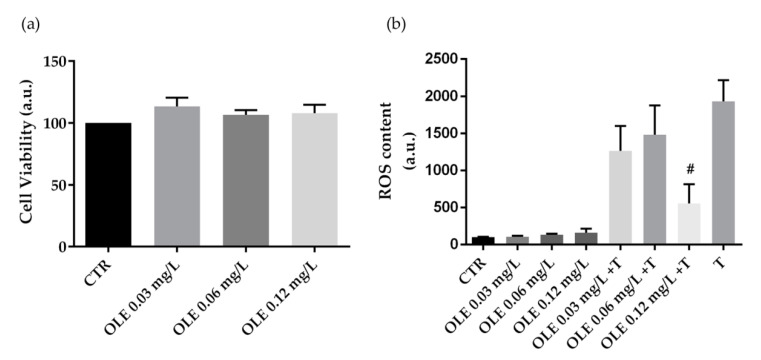
Cell viability (**a**) and ROS content (**b**) in HCT8 cells exposed to olive leaves extract (OLE). (**a**) Cells were left under basal condition or exposed to increasing concentrations (0.03 mg·L^−1^, 0.06 mg·L^−1^, and 0.12 mg·L^−1^ of phenols) of OLE and were stained with crystal violet solution. Data are shown as means ± S.E.M. of 3 independent experiments and analyzed by one-way ANOVA followed by Dunnett’s multiple comparisons test. (**b**) ROS content was measured using dihydrorhodamine-123 fluorescence in cells treated as previously described. As a positive control, cells were treated with tert-butyl hydroperoxide (tBHP). Data are shown as means ± S.E.M. of 3 independent experiments and analyzed by one-way ANOVA followed by Dunnett’s multiple comparisons test. (# *p* < 0.05 vs. tBHP).

Similar evaluations were performed in cells treated with OLE. Specifically, compared to CTR, treatment with OLE does not alter cell viability at the used concentrations (Figure 3a). Moreover, compared to the positive control (tBHP), HCT8 cells co-incubated with tBHP and OLE at 0.12 mg·L^−1^ of phenols displayed a significant reduction in ROS content. Incubation with the OLE alone, at increasing concentrations (0.03 mg·L^−1^, 0.06 mg·L^−1^, and 0.12 mg·L^−1^ of phenols) does not alter ROS content (Figure 3b).

## 4. Discussion

Extra virgin olive oil (EVOO) consumption displays protective effects on human health [26,27]. Acute intake of EVOO significantly ameliorates insulin sensitivity and glycemia in patients with metabolic syndrome by modulating the expression of genes involved in inflammation, metabolism, and cancer [28]. Furthermore, in high-fat-diet fed rats, administration of polyphenol-enriched olive oil improved insulin sensitivity, lipid profile, and interfered with inflammatory pathways [29]. Several compounds of EVOO, indeed, can modulate the NF-kB signal transduction pathway and decrease oxidative stress [30]. Importantly, the volume of wastes derived from the olive oil industry represents a worrying issue. While olive pomace can be treated to produce olive pomace oil or biomasses for fuel generation, olive mill wastewater represents a serious environmental matter and the disposal costs are very high. Notably, it has been reported that olive mill by-products and wastes can still contain numerous bioactive and valuable compounds useful in the pharmaceutical, food, and cosmetic industries. It is well established that the phenolic composition within the oil and its derivative by-products is complex and it is related to olive variety, the maturation stage, and seasons [31,32].

Oleuropein, which is found in olives, olive leaves, and oil can be degraded during olive oil production. Hydroxytyrosol and tyrosol are the main degradation products of oleuropein displaying several biological activities. Among the considered by-products, OLs are the main source of oleuropein, as reported in Table 1, since this waste is not subjected to the processes involved in oleuropein degradation during the olive oil production [33,34]. In pancreatic cancer cells, dose-dependently treatment with oleuropein or hydroxytyrosol significantly reduced cell viability and proliferation [32]. Moreover, oleuropein administration promotes apoptosis by upregulating the p53 signal pathway in breast and colon cancer cells [34,35]. Verbascoside is one of the most abundant phenolic compounds in the OLE; this compound displays beneficial effects for its antioxidant and anti-inflammatory and antitumor actions beyond the neuroprotective properties [35]. In the present report, incubation with an increasing concentration of extracts obtained from olive by-products and wastes does not alter cell viability which might be due to the low concentrations that were used. However, only the treatment with the extract obtained from the olive pomace, used at the highest concentration, caused a significant reduction in cell viability as assessed by the crystal violet assay. By comparing the extract composition of the OP with the ones of the other extracts, we found that OP extract contains higher amounts of vanillic acid and luteolin-7-glucoside. In colon HCT116 cells, exposure to vanillic acid induced G1 phase arrest and inhibited cell proliferation, possibly by inhibiting the expression of HIF-1, mTOR, and Raf/MEK/ERK signal transduction pathways [31]. Furthermore, luteolin-7-glucoside reduced keratinocyte proliferation by inhibiting IL22/STAT3 signaling [33]. Therefore, exposure to high levels of vanillic acid and luteolin-7-O-glucoside may explain the observed reduction of HCT8 cell survival exposed to the highest concentration of the OP extract.

Olive oil and olive by-products are thus a natural source of antioxidant compounds and their beneficial effects have been tested in several in vitro and in vivo experimentations [36,37,38]. Interestingly, it has been proposed that the concentration of polyphenols in the intestinal tract is higher than elsewhere because phenols can reach the colon directly or through the bile. However, relevant variability within volunteers has been also documented, which might be due to the difference in the expression level of metabolizing enzymes and to a different microbiota profile [39]. Recent reports revealed that selective phenols can interfere with oxidative and inflammatory pathways. Specifically, in a mouse model of acute colitis, administration of oleuropein-loaded lipid nanocarriers significantly reduced TNF-α release and intracellular ROS [40] strongly suggesting that phenols in the extracts may be considered valuable compounds for pharmaceutical applications. Here, with the same phenolic concentration, the extract derived from the olive wastewater displayed a higher antioxidant activity compared with the extracts derived from olive pomace and leaves. These responses might be due to the high concentration level of hydroxytyrosol and tyrosol detected in the olive mill wastewater [41]. Among phenolic compounds isolated from the olive tree, hydroxytyrosol is the most powerful antioxidant followed by oleuropein, caffeic acid and, tyrosol [42]. Conversely, at a high concentration level, antioxidants can react with molecular oxygen thereby acting as pro-oxidants [43]. Impairing antioxidant cellular defense and inducing the generation of reactive species may be a useful strategy to remove, and kill cancer cells. In human colon cancer DLD1 cells, hydroxytyrosol induced cell apoptosis, and the activation of PIPK3/AKT pathway through ROS production [44]. Accordingly, incubation with the only extract obtained from OWW, at the highest concentration, significantly increased the generation of ROS, which might be useful to activate specific intracellular signals leading to cell death as already shown by others [44,45]. Furthermore, we found that at the highest concentration level, incubation with the extract obtained from OP reduced cell viability. By contrast, treatment with phenols at a lower concentration does not exert a cytotoxic effect but it can reduce the ROS production induced by the synthetic oxidant compounds tBHP. Together, these findings suggest that phenols in the olive by-products can tightly modulate the intracellular ROS content that can act as signaling molecules cross-linking several intracellular pathways modulating cell viability.

## 5. Conclusions

This study underscores the crucial importance of the chemical and functional characterization of olive by-products. Using the ileo-carcinoma cell line HCT8 cells we found differential effects of the extracts on ROS generation and cell viability even though all extracts contain an enriched fraction of polyphenols. Therefore, further investigations would be needed to describe and identify the potential role and the possible applications and use of the bioactive compounds in the olive wastes in the pharmacological and food industry.

To conclude, according to the guiding principles of the circular economy, this study highlights and underlines the possibility of valorizing olive by-products and wastewater as they contain numerous bioactive compounds that individually or synergistically can bring beneficial effects to human health.

## Figures and Tables

**Table 1:** Total phenol content (TPC, mg GAE mL^−1^ for OWW and OP; mg GAE g^−1^ for freeze-dried OLE), antioxidant activity (µmol TE mL^−1^ for OWW and OP; µmol TE g^−1^ for OLE) and phenolic profile (mg·mL^−1^ for OWW and OP; mg·g^−1^ for OLE) of phenolic extracts.

Parameter	OWW	OP	OLE
TPC	8.61	4.46	190
ABTS	3579	451	780
Hydroxytyrosol	1.43	0.23	nd
Tyrosol	0.25	0.16	3.61
Caffeic acid	0.07	0.01	nd
Apigenin-7-*O*-glucoside	0.03	0.03	6.92
Vanillic acid	0.01	0.03	nd
Oleuropein	0.02	0.09	137
Verbascoside	nd	nd	20
Luteolin-7-*O*-glucoside	0.008	0.012	3.14
Rutin	nd	nd	3.83

GAE: gallic acid equivalents; OWW: olive mill wastewater; OP: olive pomace; TE: Trolox equivalents; OLE: olive leaves extract.
